# An analysis of optimal fertigation implications in different soils on reducing environmental impacts of agricultural nitrate leaching

**DOI:** 10.1038/s41598-020-64856-x

**Published:** 2020-05-08

**Authors:** Nasrin Azad, Javad Behmanesh, Vahid Rezaverdinejad, Fariborz Abbasi, Maryam Navabian

**Affiliations:** 1 Department of Water Engineering, Urmia University, Urmia, Iran; 2 Department of Water Engineering, Urmia University, Urmia, Iran; 3 Department of Water Engineering, Urmia University, Urmia, Iran; 4 Agricultural Engineering Research Institute, Agricultural Research, Education and Extension Organization (AREEO), Karaj, Iran; 5 Department of Water Engineering, Faculty of Agricultural Sciences, University of Guilan, Rasht, Iran

**Keywords:** Environmental impact, Agroecology, Hydrology, Engineering

## Abstract

Excessive and incorrect use of nitrogen (N) fertilizers in agriculture leads to high nitrate leaching to groundwater and harmful effects on the environment. The main objective of this research was to optimize the N fertigation scheduling for a surface micro-irrigation system in different soils. N uptake by corn and its losses were investigated for two fertigation scheduling scenarios including regional recommendation scheduling with three fertigation events and a weekly application schedule. The fertigation scheduling was then optimized to achieve both environmental objectives (minimizing nitrate losses) and corn N requirements (maximizing N uptake sufficiency). For this purpose, the HYDRUS-2D model, simulating water flow and N transport in soil, was linked to an optimization algorithm. In both scenarios, N uptake by plant was not adequate at different stages of growth in all three soil types, especially in the sandy loam soil. Optimization produced a decrease in nitrate leaching and an increase in N uptake as well as fully supplied plant requirements at different stages of corn growth. Optimization framework presented in this study and optimum fertigation scheduling in various soil textures can be applicable as a guideline for operators of micro-irrigation systems which reduce nitrate leaching and increase N uptake sufficiency.

## Introduction

Out of all human activities, agriculture is the largest consumer of water. Additionally, drainage water from agricultural activities impairs the quality of surface and groundwater resources by leaching agricultural chemicals (such as pesticides, nutrients, and salts) from the root zone of plants. Unsuitable water and fertilizer management has often induced irreparable environmental damage. Agriculture not only needs to continue producing food but should at the same time also consider environmental issues.

Widespread use of nitrogen (N) fertilizers by farmers and high leachability of nitrate results in considerable concentrations of nitrate in groundwater. Several factors affecting agricultural nitrate leaching and groundwater contamination have been identified by researchers, including fertilizer levels, manure management, crop cultivation practices, soil texture, precipitation surpluses, and others^1,2^. However, it should be possible to reduce the risk of groundwater contamination by nitrate without decreasing the crop yield by creating the balance between the N crop requirements and its efficient use by optimizing management strategies for water and N fertilizer applications in the field^3,4^. There have been many researchers all over the world who have recognized the importance of this issue and have investigated various fertilizer management practices^5–11^.

Different strategies for water and fertilizer management in agriculture have recently been investigated using numerical models simulating variably-saturated water flow and solute transport, as well as root water and nutrient uptake in soils. The use of calibrated and validated numerical models, rather than time-consuming and expensive field experiments, dramatically expanded the possibility of analyzing a large number of different management strategies for irrigation and fertigation. The HYDRUS-1D and HYDRUS (2D/3D) models^12^ have been widely used by many researchers to study different fertigation strategies. Gärdenäs *et al*. modeled the effect of the soil type and the fertigation strategy on nitrate leaching in four different micro-irrigation systems using HYDRUS-2D^13^. They showed that fertigation at the end of an irrigation cycle, compared to fertigations at the beginning or in the middle of an irrigation cycle, decreased nitrate leaching. Similarly, Hanson *et al*. used HYDRUS-2D to investigate the distribution of N in the soil and nitrate leaching for different durations of nutrient applications and various application times and concentrations^14^. Ajdary *et al*. showed that nitrate leaching could be minimized even for shallow-root crops by selecting appropriate emitter discharge, irrigation duration, and irrigation frequency^15^. Ramos *et al*. simulated water flow and N transport using HYDRUS-2D and indicated that high nitrate uptake occurred when the number of fertigation events was large, and the amount of applied fertilizer in each event was small^16^.

Phogat *et al*. used HYDRUS (2D/3D) to investigate water and nitrate dynamics in a lysimeter with an orange tree under drip irrigation during 29 days^17^. The results showed that the nitrate uptake efficiency was relatively high when fertigation was conducted at the end of a daily irrigation cycle (5 irrigation pulses) or spread over an entire duration of irrigation (compared to its application early or in the middle of a daily irrigation cycle). In another study, Phogat *et al*. simulated seasonal movement of water and nitrate under a drip-irrigated orange tree and investigated various management options to reduce nitrate leaching^18^. Karandish and Šimůnek evaluated the effects of 11 irrigation levels and 8 N fertilization rates on the water and N dynamics and the yield of maize under drip irrigation^19^. Their results showed that the combination of irrigation replacing 70% of potential evapotranspiration (*ET*) and the N fertilization of 200 kg ha^−1^ under partial root-zone drying conditions was the most efficient N-managed and water-saving irrigation strategy. Finally, Jeong and Bhattarai investigated the effects of alternative N fertilization management on nitrate losses and crop yields^20^. They showed that for N fertilizer rates of 156 and 150 kg ha^−1^, instead of the new recommendation by the Illinois nutrient loss reduction strategy (193 kg ha^−1^), nitrate losses were reduced by 10.3% and 29.8% while corn yields decreased by only 0.3% and 1.9%, respectively.

Contrary to earlier studies, in which nitrate losses and uptake were compared for a limited number of scenarios, the use of optimization methods along with simulation models allows for a wide range of different designs and management parameters to be simultaneously investigated and for an optimal management strategy of irrigation and fertigation to be selected. Kandelous *et al*. used an optimization model for optimum irrigation of alfalfa in subsurface drip irrigation^21^. A new optimization method for optimizing design and management parameters of fertigation has been developed in our previous research^22^. The main objective of this current study is to use this optimization process to optimize fertigation scheduling for a surface micro-irrigation system for different soil types. Developed fertigation scheduling for different soil types can be utilized by operators of micro-irrigation systems around the world to optimize fertilizer applications during the growing season according to plant requirements while simultaneously reducing environmental effects of nitrate leaching.

## Materials and Methods

### Micro-irrigation system

**Figure 1:**
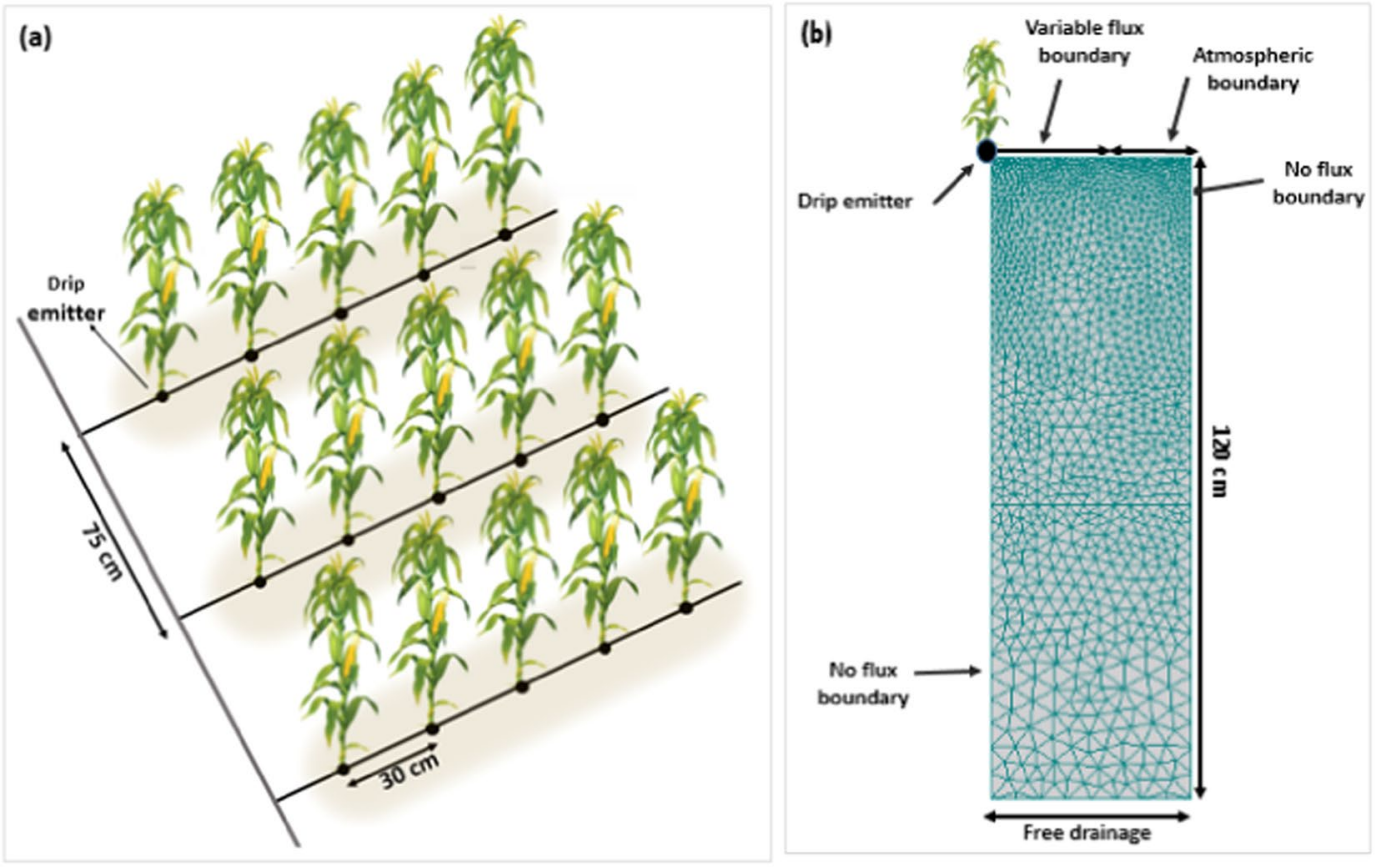
Layout of the driplines and plants (**a**) and the conceptual geometry and boundary conditions in the HYDRUS-2D simulations (**b**).

This research carried out based on experiments and measurements conducted in 2016 on a corn field with surface drip irrigation system in the location of Urmia University, Iran (detailed given by Azad *et al*.^22^). In the mentioned study, the HYDRUS-2D model and the proposed optimization algorithm were calibrated using field data collected by the authors. N plant uptake and leaching were simulated in this study in three different scenarios of N fertilizer applications for corn in a system with surface drip- irrigation. All simulations were carried out for three different soil types of silty clay (C), loam (L), and sandy loam (SL). The common corn variety grown in the northwest of Iran-Urmia plain was considered. The growth period of this variety of corn is 16 weeks, and a typical planting layout is shown in Fig. 1a.

**Figure 2:**
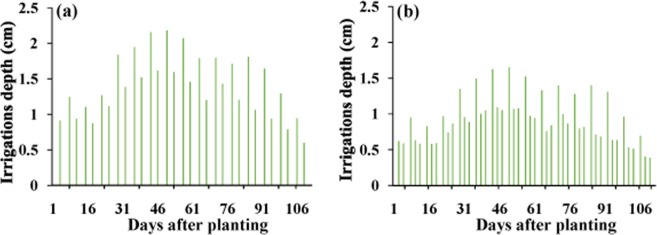
Irrigation depths for (**a**) silty clay and loamy soil (2 irrigation events per week) and (**b**) sandy loam soil (3 irrigation events per week) scenarios.

Irrigation was considered twice a week in the silty clay and loamy soils and three times a week in the sandy loam soil (Fig. 2) at the irrigation flow rate of 4 L h^−1^. Irrigation intervals choosing was based on the maximum allowed irrigation intervals according to water holding capacity of the soil and preventing water deep percolation^23^. Potential evapotranspiration of corn was calculated to determine the water irrigation depth and the duration of irrigation in each irrigation event. Total amount of irrigation water was constant in all soils. Potential crop evapotranspiration (*ET*_*p*_) was calculated using the reference crop evapotranspiration (*ET*_o_) based on the FAO Penman-Monteith equation and the dual crop coefficient approach^24^. Meteorological data required to calculate *ET*_o_ were obtained from the meteorological station at the study area. The corn N requirement, based on local recommendations, was 113.5 kg ha^−1^ N. Therefore, 334 kg ha^−1^ of ammonium nitrate fertilizer was considered for the growing season.

### Simulation model

The HYDRUS (2D/3D) model^12,25^ was used in this research to simulate water flow, solute movement, plant root growth, and root water and nutrient uptake in a two-dimensional soil profile. This program numerically solves the Richards^26^ equation for variably-saturated water movement and a convection-dispersion equation for solute transport in soil using the Galerkin finite element method.

A 2D-vertical plane (two-dimensional transport domain) with a width of 37.5 cm and a depth of 120 cm was defined in the HYDRUS-2D model (Fig. 1b). A strip wetting pattern along the driplines was represented in the model using a time-variable flux boundary condition on the left side of the soil surface, through which water and solutes entered the soil profile during irrigation/fertigation events. The third-type Cauchy boundary condition was used in this area to allow the entry of solutes at the soil surface during fertigation events. The atmospheric boundary condition (with evaporation) was specified on the rest of the soil surface.

Root growth and root densities in lateral and vertical directions were simulated using a recently developed computational module of HYDRUS-2D^22,27,28^. In this module, the dynamic rooting depth can be calculated as follows^29^:

1 $${L}_{R}(t)={L}_{m}{f}_{r}(t)$$

where ${L}_{R}(t)$ is the root length at any time (depth: $Z(t)$ and radius: $X(t)$, *L*_*m*_ is the maximum root length (maximum depth: *Z*_*m*_ and maximum radius: *X*_*m*_) and *t* is days after planting. In this equation, *f*_*r*_(*t*) is a dimensionless root growth function. This function is calculated using the classical Verhulst-Pearl logistic growth equation:

2 $${f}_{r}(t)=\frac{{L}_{0}}{{L}_{0}+({L}_{m}+{L}_{0})\exp (-rt)}$$

where *L*_0_ is the initial value of the rooting depth (recommended value=1 cm) and *r* is the growth rate (T^−1^). The growth rate, *r*, is calculated either from given data of the rooting depth at a specific time or from the assumption that 50% of the rooting depth is reached after 50% of the growing season. The second approach was used in this study. When a variable rooting depth is considered, the spatial distribution of roots must be described using either the Vrugt^30,31^ or Hoffman and van Genuchten^32^ functions. The Vrugt’s root distribution function was used to simulate both the vertical and horizontal growth of the roots.

The maximum depth and radius of the corn roots were considered to be 60 and 35 cm^6,33^, respectively. Similarly, as in Wang *et al*.^33^, the parameters defining the maximum root water uptake intensity in vertical and horizontal directions (z^*^ and x^*^) were selected to be 10 and 0 cm, respectively, and the shape coefficients *p*_*x*_ and *p*_*z*_ were set to 1.0. The reduction of root water uptake due to the water stress was described using the macroscopic approach of Feddes *et al*.^34^ with specific corn coefficients from the HYDRUS-2D database^35^.

HYDRUS-2D uses the van Genuchten-Mualem functions^36^ to describe soil hydraulic properties, i.e., retention curves and hydraulic conductivity functions. The parameters for these relationships (i.e., the residual water content *θ*_*r*_, the saturated water content *θ*_*s*_, the van Genuchten shape parameters [*α*, *n*, and *l*], and the saturated hydraulic conductivity *K*_*s*_) were taken for loam and sandy loam from Carsel and Parish^37^ and for silty clay from the Rosetta database^38^, similarly as done by Gärdenäs *et al*.^13^. The soil hydraulic parameter values are listed in Table 1.

**Table 1:** Soil hydraulic properties of selected soil types^11,33,34^.

Textural class	*θ*_*r*_ (cm^3^ cm^−3^)	*θ*_*s*_ (cm^3^ cm^−3^)	*α* (cm^−1^)	*n* (-)	*K*_*s*_ (cm day^−1^)
Silty clay, C	0.111	0.481	0.0162	1.32	9.61
Loam, L	0.078	0.43	0.036	1.56	24.96
Sandy loam, SL	0.065	0.41	0.075	1.89	106.1

The convection-dispersion equation and the first-order decay chain were used to simulate the transport and transformations of N species, respectively. These equations and their parameters are described in Šimůnek *et al*.^39^. Since ammonium nitrate was considered as a fertilizer, ammonium adsorption to the soil particles and nitrification (NH_4_^+^ transformation into NO_2_^−^ and then further into NO_3_^−^) were considered as the main reaction processes. The distribution coefficient (*K*_*d*_) for ammonium sorption and the first-order rate constants for nitrification of ammonium to nitrate in the liquid and solid phases (*µ*′_*w*_ and *µ*′_*s*_, respectively) were specified using parameters reported in the literature^13,14,16,33,40,41^: 3.5 cm^3^ gr^−1^, 0.2 day^−1^, and 0.2 day^−1^ for *K*_*d*_, *µ*′_*w*_, and *µ*′_*s*_, respectively. The longitudinal dispersivity (*ε*_*L*_) and the transverse dispersivity (*ε*_*T*_) were set to be one-tenth of the soil depth and one-tenth of *ε*_*L*_, respectively^16^. The initial concentrations of ammonium and nitrate were set to a uniform zero concentration similar to the research of Gardenas *et al*.^13^. Similarly as in many other studies^14–16,41,42^ mineralization and immobilization were neglected. Furthermore, in drip irrigation, the process of denitrification can be neglected due to unsaturated and aerobic conditions in the soil^41^. Similar to the present study, in the research of Gardenas *et al*.^13^ denitrification losses were ignored in silty-clay, loam and sandy loam soils in the micro-irrigation system. Finally, similar to the study of Ramos *et al*.^16^, unlimited passive nutrient uptake^43^ was considered for N species. The N balance components, including accumulation and leaching, were evaluated for the root zone 65 cm deep.

### Fertigation scenarios and optimization of fertigation scheduling

In the first step, water flow, water uptake by plants, plant growth, solutes transport, nitrate leaching, and N uptake by plants were investigated by considering two different fertigation scenarios with fixed application frequencies during the growing season. In the first fertigation scenario, fertilizer applications were divided into three splits, which are used by local farmers. In this scenario, 50, 25, and 25% of the total N fertilizer was applied at the beginning of the growing season, at the knee stage, and at the tasselling stage, respectively. In the second fertigation scenario, the fertilizer was applied weekly throughout the entire growing season. In both cases, the fertilizer was applied at the end of the irrigation event (before providing the opportunity to wash the pipes and emitters after the fertilizer application). The duration of the fertilizer application in each fertigation event was based on the minimum allowed period of the application to meet the criteria of *EC* < 3 dS m^−1^ of the irrigation water^44^. The minimum application time was considered to be 5 minutes.

In the second step, the design and management parameters of irrigation and fertigation (including irrigation flow rate, duration and start time of fertigation and also, fertilizer amounts in each fertigation event) were optimized for three soil textural types. The objective of this optimization was to increase N uptake sufficiency and to decrease environmental contamination due to nitrate leaching. It should be noted that in addition to nitrate leaching from the soil profile throughout the growing season, nitrate accumulated in the soil profile at the end of the growing season will likely also leach from the soil profile and be transported to groundwater aquifers due to autumn and winter precipitation after crop harvesting. In fact, both nitrate leaching during the growing season and its accumulation at the end of the growing season in the soil profile should be avoided when optimizing design and management parameters of fertigation.

Optimization was done in two stages. First, the irrigation flow rate (*Q*), the start time of the fertilizer application (*T*_*start*_), and the duration of fertigation (*T*_*fer*_) were optimized for each soil type to minimize nitrate leaching. The objective function was as follows:

3 $$OF1={D}_{wp}+N{O}_{3}^{-}\_L$$

where $\,OF1$ is the objective function of the first stage of optimization, ${D}_{wp}$ is deep water percolation and $N{O}_{3}^{-}\_L$ is leached nitrate. These components are dimensionless as a fraction of the input value of water or nitrate. The optimization was carried out for a duration of one week with two or three irrigation events (depending on the soil texture) while fertigation was applied in the first irrigation event of the week. This approach allowed us to consider the effects of subsequent irrigation events (one or two) without fertigation on nitrate leaching.

Second, after determining the best combination of decision variables (i.e., *Q*, *T*_*start*_, and *T*_*fer*_), the fertilizer amounts were optimized for each fertigation event of the growing season based on the plant’s N demand at different stages of the plant growth. Besides supplying plant’s N requirements, nitrate leaching during the growing season and nitrate accumulation in the soil profile at the end of the growing season were minimized. The objective function was as follows:

4 $$OF2={S}_{diff}+N{O}_{3}^{-}\_Losses$$

where $\,OF2$ is objective function of the second stage of optimization, ${S}_{diff}$ is cumulative difference between the plant demand and uptaken N, $N{O}_{3}^{-}\_Losses$ is total leached and accumulated nitrate in the soil. The minimum allowed duration of the fertilizer application for optimized irrigation rates was set to prevent exceeding maximum irrigation water salinity (*EC*_*iw*_ < 3 dS m^−1^). Additional explanations about the objective functions, decision variables, and constraints of the optimization model during the two optimization stages are given in Azad *et al*.^22^. The Particle Swarm Optimization (PSO) method^45,46^ was employed in the optimization process. Optimization was run in MATLAB linked with the HYDRUS-2D model.

## Results and Discussion

The results of the simultaneous optimization of the irrigation flow rate (*Q*), the start time of the fertilizer injection (*T*_*start*_), and the duration of fertigation (*T*_*fer*_) to limit water and nitrate leaching from the root zone for three soil textures of silty clay (C), loam (L), and sandy loam (SL) are given in Table 2. The irrigation flow rate of 3.67 L hr^−1^ (for each dripper) and fertigation at the end of the first irrigation event (i.e., T^*^, irrigation time minus irrigation pipes’ washing time) for the duration of 0.23 T^*^ are the optimum decision parameters for the silty clay soil. Deep percolation water losses and nitrate leaching during a weekly fertilizer cycle, which includes one irrigation event with a fertigation application and one without, are minimal in this soil type for these optimum decision parameters. Optimal irrigation flow rates for the loam and sandy loam soils were 1.65 and 0.8 L hr^−1^, respectively, with fertigation at the end of T^*^ and 5 minutes duration. The optimized fertilization cycle for the loamy soil was the same as for the silty clay soil. However, for the sandy loam soil, the fertigation cycle involved a fertigation application at the beginning of the week and two subsequent irrigation events without fertigation. It is noted that the total amount of irrigation water was constant and the irrigation time was changing in the optimization process based on the variation of irrigation flow rate.

**Table 2:** Optimization results of the fertigation design and management parameters in one fertigation cycle.

Soil type	Optimized values		
	*Q*(L h^−1^)	The start time of the fertilizer application, *T*_*start*_	Duration of the fertilizer application, *T*_*fer*_
Silty Clay (C)	3.67	End of T^*^	23% of T^*^
Loam (L)	1.65	End of T^*^	Minimum limit
Sandy Loam (SL)	0.8	End of T^*^	Minimum limit

T^*^: Irrigation time minus the washing time (for ensuring the flushing of the drip lines and emitters).

**Figure 3:**
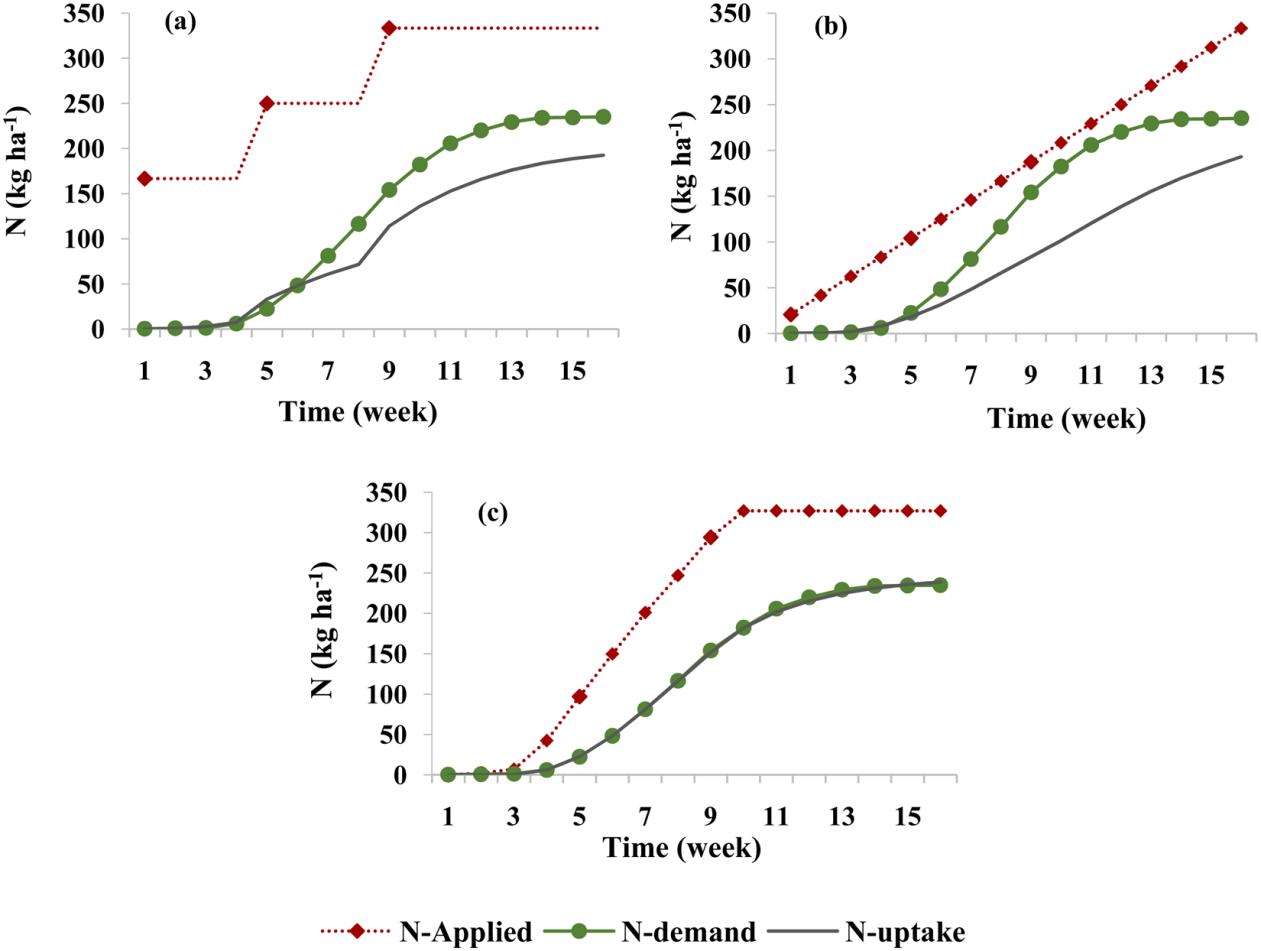
Comparison between corn N requirements during its growth and simulated N uptake in the silty clay soil for different fertilizer application schedules: (**a**) three fertilizer applications (local recommendations), (**b**) weekly fertilizer applications, and (**c**) optimized fertigation scheduling.

In the second optimization stage, after optimizing the design and management parameters of the fertigation cycle in the first optimization stage, the amounts of applied fertilizer in each fertigation event during the growing season were optimized. During this stage, the amount of nitrate leaching and its accumulation at the end of the growing season were minimized, in addition to maximizing the supply of N to meet plant requirements during different growth stages. The results of the optimization for the silty clay soil are shown in Fig. 3. This figure compares corn N requirements at different growth stages^47,48^ with simulated N uptake for three fertigation scenarios, two scenarios with fixed schedules (three fertilizer applications and weekly applications), and one with the optimized schedule.

When fertilizer was provided in three applications (Fig. 3a), plant N uptake was not adequate at different crop growth stages, and the plant was at risk of N deficiency. The analysis of N balance components for this scenario (Table 3) indicates that about 56% of the total applied N (which includes nitrate nitrified from ammonium) was taken up by the crop, while about 7% was leached during the growing season, and about 37% remained in the soil profile at the end of the growing season.

**Table 3:** Simulated components of the soil N balance in the root zone of the silty clay soil for the growing season for different fertilizer application schedules.

	Fertilizer application scheduling	Unit	N balance components					
			Applied	Nitrification	Plant uptake	Accumulated	Leached	Losses^††^
NH_4_^+^	Three splits	kg ha^−1^	74.90	73.17	1.54	0.00	0.19	0.19
		%		97.69	2.05	0.00	0.26	0.26
	Weekly applications	kg ha^−1^	75.36	71.40	1.87	1.67	0.41	2.08
		%		94.75	2.49	2.22	0.55	2.76
	Optimized scheduling	kg ha^−1^	73.49	69.85	2.98	0.00	0.65	0.65
		%		95.05	4.06	0.00	0.89	0.89
NO_3_^−^	Three splits	kg ha^−1^	258.87	73.05	186.74	123.11	22.07	145.18
		%			56.26	37.09	6.65	43.74
	Weekly applications	kg ha^−1^	259.45	72.23	186.67	131.17	13.85	145.02
		%			56.28	39.55	4.18	43.72
	Optimized scheduling	kg ha^−1^	254.92	69.46	231.36	78.38	14.65	93.02
		%			71.32	24.16	4.52	28.68

^†^A sink term for ammonium and a source term for nitrate.

^††^Total leached and accumulated N in the soil.

When fertilizer was applied in equal weekly splits during the growing season, the plant was still unable to uptake its N requirements at different stages of growth (Fig. 3b). In this scenario, low nitrate leaching (about 4%) occurred in this relatively fine-textured soil during the growing season (Table 3). However, since the applied N rate was not proportional to the plant requirements at different growth stages, the plant did not get enough fertilizer at appropriate times, and about 40% of nitrate accumulated in the root zone. Accumulated nitrate may be the main reason of groundwater contamination in fine-textured soils since it is susceptible to leaching during the rainy season. Wang *et al*. emphasized that heavy precipitation at the end of the growing season caused more deep percolation than during the growing season^33^. In the studied region, while the corn growing season is often without any rainfall, significant autumn rainfalls occur after the harvest. It is, therefore, necessary to manage and control not only nitrate leaching during the growing season, but also nitrate accumulation at the end of the growing season. As shown in Fig. 3c, optimizing the amount of applied fertilizer increases the supply of N to meet corn requirements at different stages of growth. Nitrate accumulation in the root zone decreased by increasing N uptake to about 71% of applied N and reducing nitrate losses to about 29% (Table 3).

**Figure 4:**
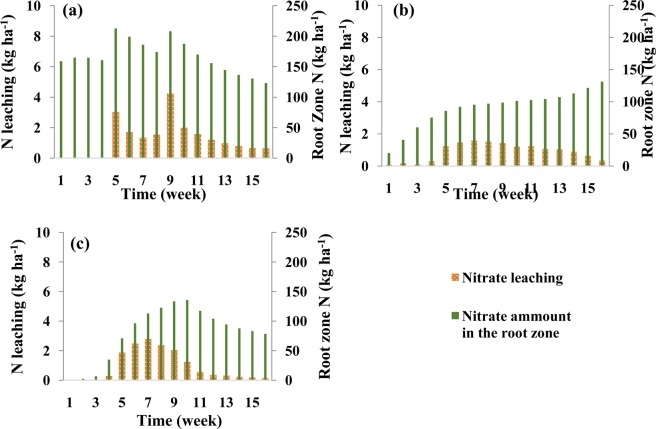
Weekly leaching of nitrate and the nitrate amount in the root zone at the end of each week in the silty clay soil for different fertilizer application schedules: (**a**) three fertilizer applications (local recommendations), (**b**) weekly fertilizer applications, and (**c**) optimized fertigation scheduling.

Figure 4 shows nitrate leaching during each week of the growing season and the amount of nitrate in the root zone at the end of each week in the silty clay soil for different fertigation scenarios. In the scenario with three N applications (Fig. 4a), nitrate accumulation in the root zone increased with the first N application at the beginning of the growing season and then remained nearly constant till the fourth week due to very low root N uptake and no leaching. The second and third N applications produced an instantaneous increase in the amount of nitrate in the root zone and then its gradual decrease due to leaching and roots uptake. In the scenario with weekly N applications (Fig. 4b), nitrate storage gradually increased during the first six weeks, then remained relatively stable during several weeks due to increased uptake by plant roots, before starting to increase again at the end of the growing season. Nitrate leaching started after about four weeks when nitrate reached the bottom of the root zone (the depth of 65 cm), but remained relatively low during the entire season. In the scenario with optimized fertigation (Fig. 4c), the accumulation of nitrate in the root zone was reduced and remained at the lowest level of all three scenarios at the end of the season, while nitrate leaching was higher than in the other two scenarios in the middle and significantly lower at the end of the growing season.

**Figure 5:**
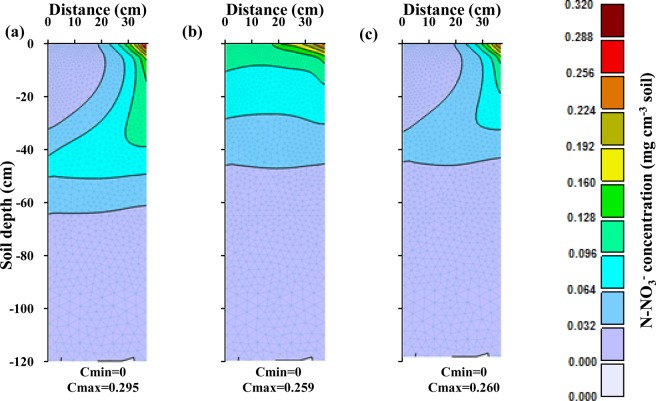
Spatial distribution of nitrate concentrations in the soil profile at the harvesting time in the silty clay soil for different fertilizer application schedules: (**a**) thee fertilizer applications (local recommendations), (**b**) weekly fertilizer applications, and (**c**) optimized fertigation scheduling. (Images were resulted from HYDRUS (2D/3D) model; version: 2.05; www.pc-progress.com).

Figure 5 shows the spatial distribution of nitrate in the soil profile of the silty clay soil at the harvest time for different fertigation scenarios. In the scenario with three fertilizer applications, significant amounts of nitrate accumulated in the root zone at the end of the growing season and leached below the root zone. In the scenario with weekly applications of smaller fertilizer amounts during each fertigation event, the nitrate front penetrated less deeply than in the scenario with three fertilizer applications. On the other hand, a significant amount of nitrate accumulated in the soil profile in this relatively fine-textured soil. In the scenario with optimized fertilizer applications, the advancement of the nitrate front was slowed in comparison with the three-application scenario. Also, in the latter scenario, the nitrate accumulation in the root zone was smaller than in previous scenarios.

**Figure 6:**
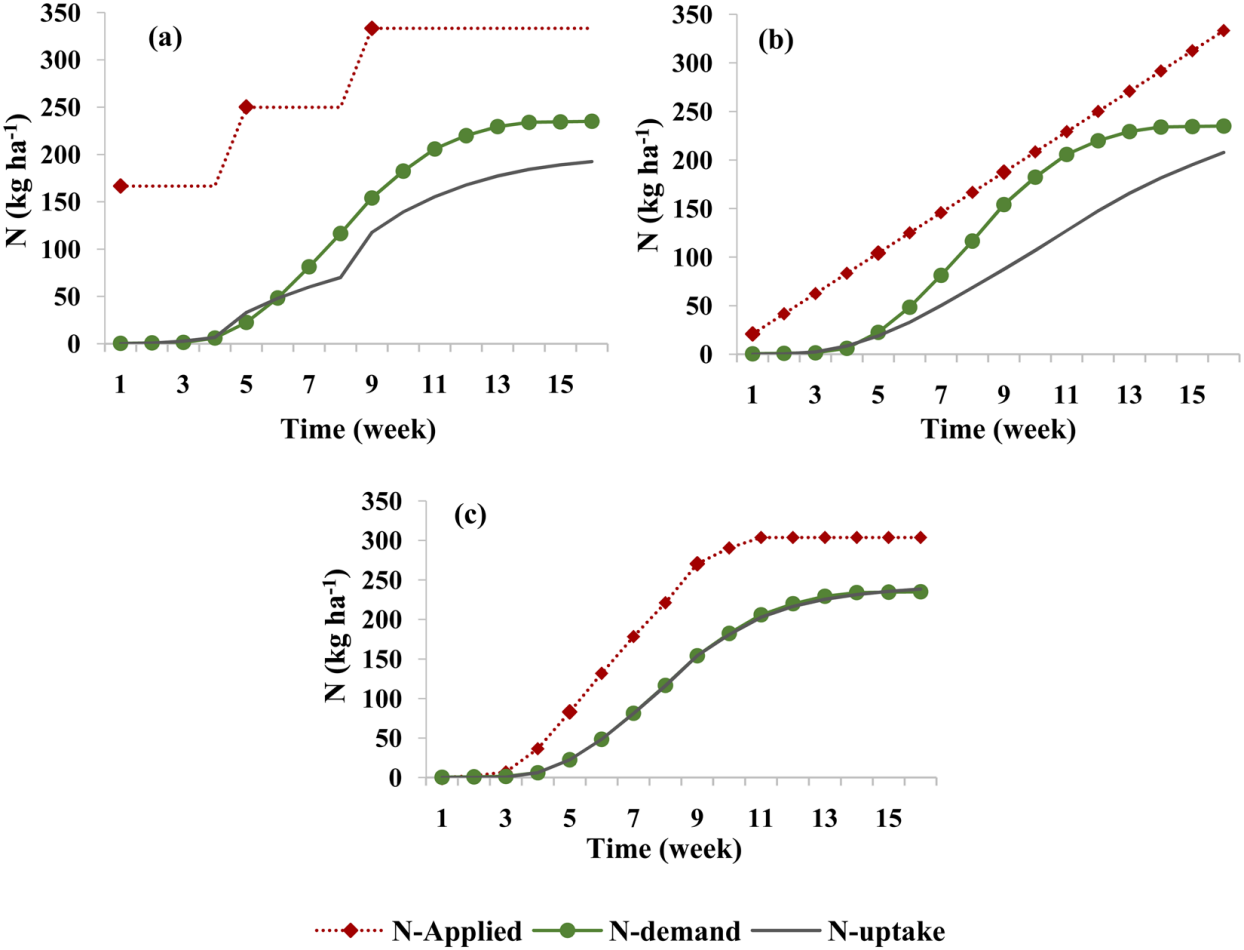
Comparison between corn N requirements during its growth and simulated N uptake in the loamy soil for different fertilizer application schedules: (**a**) three fertilizer applications, (**b**) weekly fertilizer applications, and (**c**) optimized fertigation scheduling.

A comparison of the crop N requirement with simulated N uptake in three fertigation scenarios for the loamy soil is shown in Fig. 6. Table 4 provides N mass balance components for these conditions. In the three-split scenario, about 187 kg ha^−1^ (56%), 112 kg ha^−1^ (34%), and 34 kg ha^−1^ (10%) of the total amount of applied N was taken up, accumulated, and leached, respectively. In the weekly scenario, plant uptake increased to about 61% and total losses decreased to about 40%. These results correspond to Ramos *et al*., who showed, by using two-dimensional modeling of water and N dynamics in the medium-textured soil, that nitrate uptake was higher when fertigation events were more numerous, and the amount of applied fertilizer in each event was smaller^16^. Similarly, Marinov and Marinov indicated that gradual fertilization during the growing season in the medium-textured soil reduced the potential for nitrate leaching and contamination of groundwater^49^. Other studies evaluating irrigation-fertigation systems for different soil and plant conditions also indicated lower nitrate leaching, higher N uptake, and higher crop yields for high fertigation frequency conditions^9,50^. The research of Farneselli *et al*. also showed that the high frequency of fertigation and/or irrigation could be a good strategy to increase the N uptake efficiency^5^.

**Table 4:** Simulated components of the soil N balance in the root zone of the loamy soil for the growing season for different fertilizer application schedules.

	Fertilizer application scheduling	Unit	N balance components					
			Applied	Nitrification^†^	Plant uptake	Accumulated	Leached	Losses^††^
NH_4_^+^	Three splits	kg ha^−1^	74.82	73.55	1.49	0.00	0.00	0.00
		%		98.30	1.99	0.00	0.00	0.00
	Weekly applications	kg ha^−1^	75.36	72.05	1.81	1.69	0.00	1.69
		%		95.61	2.40	2.24	0.00	2.24
	Optimized scheduling	kg ha^−1^	68.16	65.75	2.69	0.00	0.00	0.00
		%		96.46	3.95	0.00	0.00	0.00
NO_3_^−^	Three splits	kg ha^−1^	258.60	73.45	186.52	111.57	33.97	145.54
		%			56.17	33.60	10.23	43.83
	Weekly applications	kg ha^−1^	259.45	71.90	200.44	123.83	7.09	130.92
		%			60.49	37.37	2.14	39.51
	Optimized scheduling	kg ha^−1^	235.40	66.26	231.05	70.56	0.06	70.62
		%			76.59	23.39	0.02	23.41

However, as shown in Fig. 6, neither scenario with fixed applications, nor with three applications or with weekly applications, met the plant requirements at different stages of the plant growth. The scenario with optimized applications of fertilizer during the growing season in the loam soil not only supplied the required N at all times but also used a smaller amount of fertilizer (303 kg ha^−1^ instead of 334 kg ha^−1^). With this scheduling, about 231 kg ha^−1^ (77%) of the applied N were taken up by the plants, and nitrate leaching and its accumulation at the end of the growing season decreased to almost zero (0.06 kg ha^−1^; 0.02%) and about 71 kg ha^−1^ (23%), respectively.

**Figure 7:**
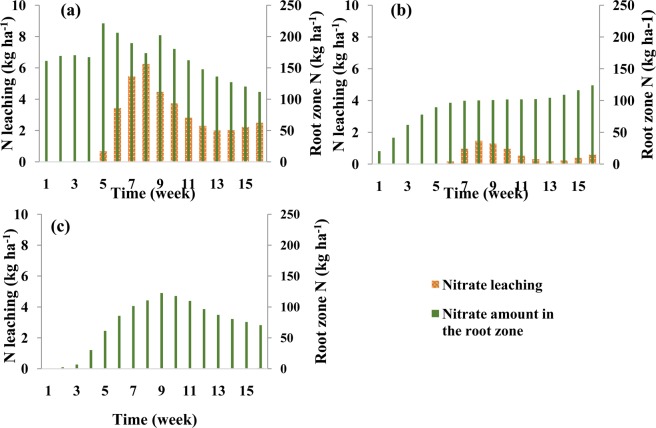
Weekly leaching of nitrate and the nitrate amount in the root zone at the end of each week in the loamy soil for different fertilizer application schedules: (**a**) three fertilizer applications, (**b**) weekly fertilizer applications, and (**c**) optimized fertigation scheduling.

Nitrate leaching during each week of the growing season and its amounts in the root zone at the end of each week in the loamy soil and for different fertigation scenarios are shown in Fig. 7. In the three-split scenario, the nitrate leaching front reached the depth of 65 cm during the fifth week when leaching below the root zone started. Nitrate leaching gradually increased until the ninth week when it started decreasing due to high nitrate uptake by the plants, and then remained about the same until the end of the growing season. Contrary to the second fertilizer application, the third application did not cause higher nitrate leaching due to the higher N requirement by the plants during this part of the growing season.

**Figure 8:**
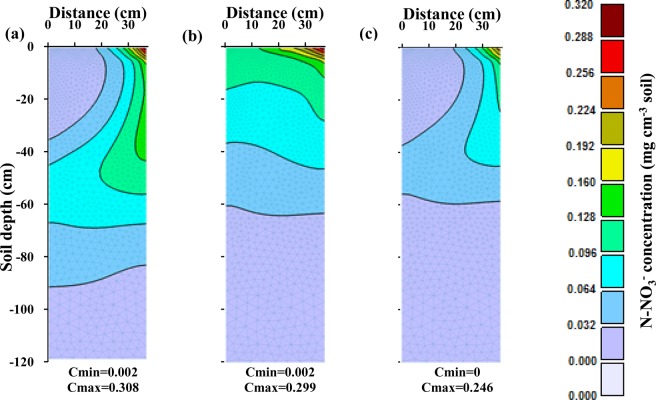
Spatial distribution of nitrate concentrations in the soil profile at the harvesting time in the loamy soil for different fertilizer application schedules: (**a**) three fertilizer applications, (**b**) weekly fertilizer applications, and (**c**) optimized fertigation scheduling. (Images were resulted from HYDRUS (2D/3D) model; version: 2.05; www.pc-progress.com).

An analysis of the nitrate spatial distribution in the loamy soil at the harvest time for different fertilizer application schedules (Fig. 8) indicates that the optimized fertigation schedule reduced nitrate leaching to practically zero and also decreased nitrate accumulation in the soil profile compared to the other fertigation scenarios.

Comparing the results for the loamy soil with those for the silty clay soil indicated that nitrate leaching in the three-split scenario was higher in the loamy soil than in the silty clay soil (Fig. 4a). In the weekly scenario, nitrate leaching in the loamy soil began later than in the silty clay soil (Fig. 4b), and the rate of leaching was also lower. N uptake by plants in the loamy soil (Table 4) was higher than in the silty clay soil (Table 3). As a result, nitrate leaching and nitrate accumulation in the root zone was lower in the loamy soil than in the silty clay soil, even though nitrate in the loamy soil leached deeper than in the silty clay soil.

**Figure 9:**
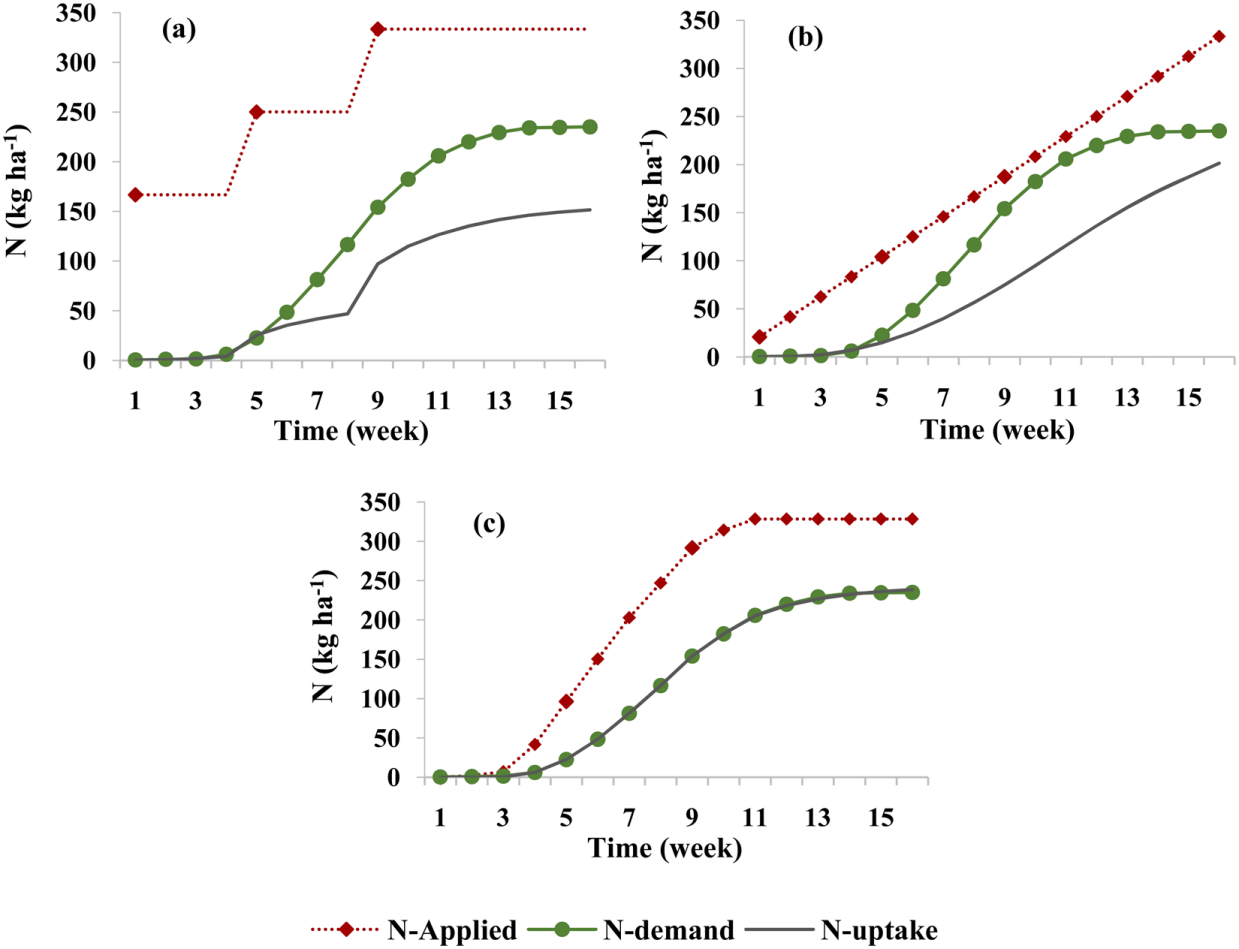
Comparison between corn N requirement during its growth and simulated N uptake in the sandy loam soil for different fertilizer application schedules: (**a**) three fertilizer applications, (**b**) weekly fertilizer application, and (**c**) optimized fertigation scheduling.

**Figure 10:**
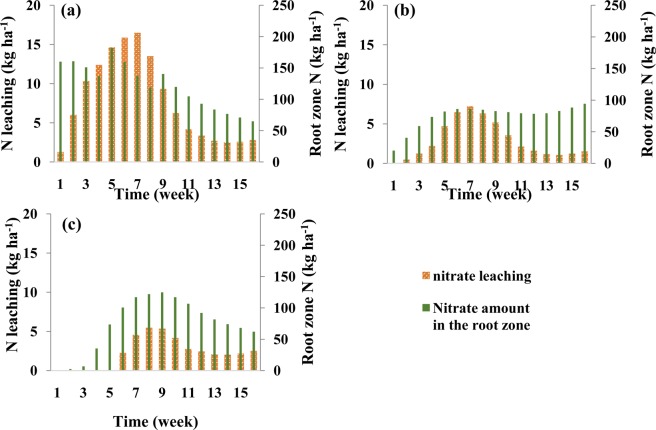
Weekly leaching of nitrate and the nitrate amount in the root zone at the end of each week in the sandy loam soil for different fertilizer application schedules: (**a**) three fertilizer applications, (**b**) weekly fertilizer applications, and (**c**) optimized fertigation scheduling. Note a different scale (double) on a vertical axis representing N leaching than in Figs. 6 and 9.

In the sandy loam soil, the amount of N taken up by the plant in the three-split scenario was significantly smaller than both plant requirements or the amount taken up in other soil textures (Fig. 9). This was caused mainly by higher leachability of nitrate in this soil texture (Fig. 10), as applied nitrate rapidly leached below the root zone in the three-split scenario. In the scenario with weekly fertilizer applications during the growing season, N uptake increased (compared to the three-split scenario) from 44% to 58% and leaching decreased from 37% to 13% (Table 5). Similar results were obtained by Rajput and Patel, who indicated that increasing the fertigation frequency in a sandy loam soil increased the yield of onions^10^. However, nitrate accumulation in the soil profile for the weekly fertilizer applications increased by about 9% in comparison with the three-split scenario. This may be related to the fertilizer deficit during a time of higher plant N requirements, as well as to the accumulation of excess fertilizer in the root zone during low plant requirements. Accumulated nitrate in the coarse-textured soil profile has a higher susceptibility to leaching than in other textures. The optimized fertigation schedule provided an adequate supply of N to the plants (Fig. 9c), with N uptake reaching 71% of applied N (Table 5). Furthermore, the accumulation of nitrate in the soil profile at the end of the growing season decreased to about 19% and its leaching below the root zone was delayed and decreased to about 11%.

**Table 5:** Simulated components of the soil N balance in the root zone of the sandy loam soil for the growing season for different fertilizer application schedules.

	Fertilizer application scheduling	Unit	N balance components					
			Applied	Nitrification^†^	Plant uptake	Accumulated	Leached	Losses^††^
NH_4_^+^	Three splits	kg ha^−1^	75.22	74.21	1.31	0.00	0.00	0.00
		%		98.66	1.74	0.00	0.00	0.00
	Weekly applications	kg ha^−1^	75.37	72.42	1.61	1.68	0.00	1.68
		%		96.08	2.13	2.23	0.00	2.23
	Optimized scheduling	kg ha^−1^	73.40	71.27	2.55	0.00	0.00	0.00
		%		97.10	3.47	0.00	0.00	0.00
NO_3_^−^	Three splits	kg ha^−1^	258.60	75.73	147.47	64.79	122.06	186.86
		%			44.11	19.38	36.51	55.89
	Weekly applications	kg ha^−1^	258.88	74.66	194.36	94.26	44.93	139.19
		%			58.27	28.26	13.47	41.73
	Optimized scheduling	kg ha^−1^	255.33	73.46	232.16	62.04	34.59	96.63
		%			70.61	18.87	10.52	29.39

^†^A sink term for ammonium and a source term for nitrate.

^††^Total leached and accumulated N in the soil.

**Figure 11:**
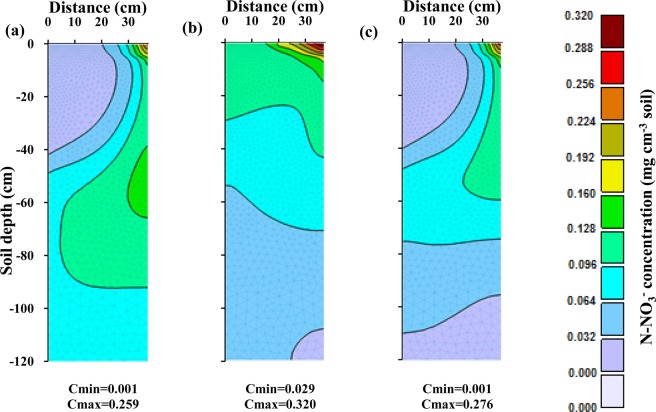
Spatial distribution of nitrate concentrations in the soil profile at the harvesting time in the sandy loam soil for different fertilizer application schedules: (**a**) three fertilizer applications, (**b**) weekly fertilizer applications, and (**c**) optimized fertigation scheduling. (Images were resulted from HYDRUS (2D/3D) model; version: 2.05; www.pc-progress.com).

Figure 10 shows nitrate leaching and nitrate storage in the root zone at the end of each week in the sandy loam soil. This figure indicates that significant nitrate leaching occurred in this coarse-textured soil in all scenarios. In the three-split scenario, applied nitrate rapidly reached the depth of 65 cm, and its leaching increased due to subsequent irrigations. In the scenario with weekly fertilizer applications, nitrate reached the bottom of the root zone at lower concentrations and thus its leaching was smaller. Since very little fertilizer was applied in early weeks in the optimized fertilizer schedule, nitrate leaching from the root zone was delayed (Fig. 10c). Although in this case nitrate leaching and nitrate accumulation was lower than in the other scenarios, nitrate leaching could not be fully prevented in this coarse-textured soil compared to the other two textures even when the fertilization schedule was optimized. But in this soil, like the other two soils, the optimized fertigation schedule increased the supply of N to meet corn requirements at different stages of growth. Figure 11 also shows a slower nitrate front advance in the soil profile in the optimized fertilization scenarios compared to the fixed scenarios.

**Figure 12:**
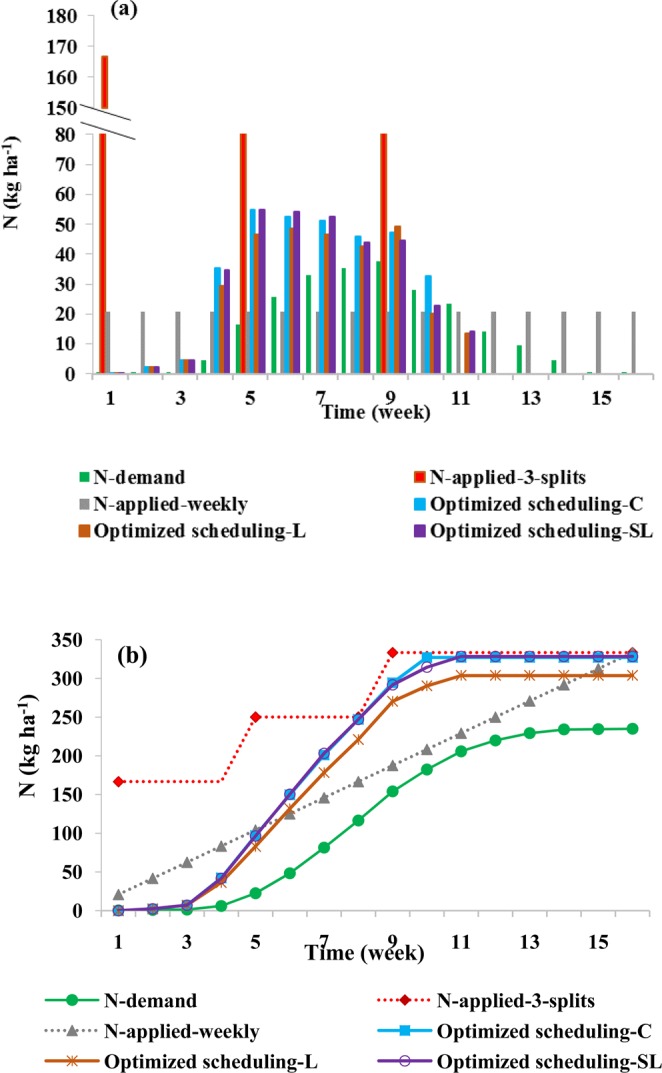
Weekly (**a**) and cumulative (**b**) N fluxes, including crop N demand (N-need), N applied in 3 splits, N applied weekly, and in optimized N amounts. Optimized applications (Optimized scheduling) are for the silty clay (C), loam (L), and sandy loam (SL) soils.

Various weekly and cumulative N fluxes, including crop demand, and three-split, weekly, and optimized N applications for three soil types, are summarized in Fig. 12. There are not large differences between optimized N applications (max differences were less than 10 kg ha^−1^ week^−1^) for different soil types (Fig. 12a). Optimized N applications for all three soil types are generally very small during the first three weeks when the crop demand is still minimal, increase in week four, and reach their maximum values at week 5. They remain more or less constant (slightly increasing) through week 9, and then drop off until they are zero at week 12. The crop N demand (approximately a half sine wave with a maximum of about 40 kg ha^−1^ week^−1^) lags by about two to four weeks behind the N applications, reaching the maximum value at week 9.

A comparison of the results of the optimized fertilization scenario in three different soil types shows that the higher nitrate losses in the silty clay soil can be explained mainly by a higher nitrate accumulation in the root zone (Table 3) and in the sandy loam soil by higher nitrate leaching (Table 5). In fact, optimization eliminates the disadvantages of both three-split scenario (with a high nitrate leaching problem, especially in sandy loam soil) and weekly scenario (with a high nitrate accumulation problem, especially in silty clay soil). Nitrate losses in the loamy soil were smaller than in the other two textures (Table 4). It should also be emphasized that a smaller amount of fertilizer had to be applied in the loamy soil to fully supply corn N requirements than in the other two soils (Fig. 12b). Furthermore, the rate of nitrate losses reduction and increasing nitrate uptake in optimized fertilization scenario compared to other two scenarios was higher in sandy loam soil than in two other soil textures. In fact, optimization in a coarse-textured soil can reduce its losses to a fine-textured soil level.

## Conclusions

The results of this study showed that the optimized fertigation schedules for three soil textures increased N uptake and provided sufficient N supply during different stages of the corn growth, as well as reduced nitrate losses (its leaching and accumulation at the end of the growing season) in comparison with scenarios involving either regional recommendations for fertilizer applications or uniform weekly fertilizer applications. Accumulated nitrate at the end of the season is susceptible to leaching during the rainy season and can contaminate groundwater after harvesting. Considering the necessity of supplying the plant’s N requirement to maintain its yield as well as reducing nitrate pollution in groundwater due to inappropriate use of N fertilizers, fertigation design and management parameters need to be optimized for different soil types and crops. Therefore, the optimum fertigation scheduling presented in this study in various soil textures can be important and applicable. Guidelines can be developed based on the results of this study to help operators of micro-irrigation systems to better design and manage fertigation systems in similar conditions as used in this study. Furthermore, a similar optimization framework can be used for other conditions, involving different soil textures, different crops, different meteorological conditions, and different irrigation systems.

## Data Availability

The data that support the findings of this study are openly available.
